# Supplementary material for: How clustered protocadherin binding specificity is tuned for neuronal self-/nonself-recognition
Source: eLife. 2022 Mar 7;11:e72416. doi: 10.7554/eLife.72416 (PMC8901172; doi:10.7554/eLife.72416)
Supplement: Figure 2—source data 1. [file elife-72416-fig2-data1.docx]

| Protein | Oligomeric State | Dissociation Constant,  K_D_ (μM) |
| --- | --- | --- |
|  |  |  |
| *Trans-interacting fragments* |  |  |
| α4_1–5_ | Dimer | 5.0 ± 0.80 |
| α7*_1–5_ | Dimer | 2.91 ± 0.55 |
| α12_1–5_ | Dimer | 34 ± 2.8 |
| β6*_1–4_ | Dimer | 16.3 ± 2.1 |
| β8*_1–4_ | Dimer | 24.0 ± 0.43 |
| γA1*_1–4_ | Dimer | 13.3 ± 0.93 |
| γA4*_1–4_ | Dimer | 45.3 ± 1.52 |
| γA8*_1–4_ | Dimer | 30 ± 1.5 |
| γA9*_1–5_ | Dimer | 8.61 ± 0.35 |
| γB2*_1–5_ | Dimer | 21.8 ± 0.21 |
| γB4_1–5_ | Dimer | 38 ± 0.33 |
| γB5* _1–4_ | Dimer | 79.1 ± 4.3 |
| γB5_1–4_-AVI | Dimer | 50 ± 0.4 |
| αC2*_1–4_ | Dimer | 20.6 ± 1.19 |
| γC3*_1–4_ | Dimer | 115 ± 1.49 (K_i_/K_D_ = 1.57) |
| γC4_1–4_ | Monomer / Very weak dimer | > 500^†^ |
| γC5*_1–5_ | Dimer | 100 ± 4.33 |
|  |  |  |
| *Trans mutants* |  |  |
| α7_1–5_ L301R | Weakly dimeric | 490 ± 57 |
| γA8_1–4_ I116R* | Monomer | N/A |
| αC2*_1–3_ | Dimer | 242 ± 0.1 (K_i_/K_D_ = 1.48) |
| β_1–4_ R41N | Dimer | 160 ± 0.38 |
| β_1–4_ S117I | Dimer | 72 ± 34 |
| β_1–4_ L125P | Dimer | 150 ± 20 |
| β_1–4_ E369K | Dimer | 23 ± 2.8 |
| β_1–4_ Y371F | Dimer | 39 ± 5.6 |
| β_1–4_ R41N/S117I | Precipitate | N/A |
| β_1–4_ R41N/E369K | Dimer | 41 ± 0.69 |
| β_1–4_ S117I/L125P | Dimer | 68 ± 5.1 |
| β_1–4_ R41N/S117I/L125P | Weak dimer | 350 ± 11 |
| β_1–4_ R41N/S117I/E369K | Dimer | 32 ± 0.95 |
| β_1–4_ R41N/S117I/Y371F | Dimer | 18 ± 0.11 |
| β_1–4_ R41N/S117I/L125P/E369K/Y371F | Dimer | 63 ± 11 |

#### Figure 2—source data 1. Sedimentation equilibrium analytical ultracentrifugation data for *trans* SPR reagents

* Previously published data (Rubinstein et al., 2015; Goodman et al., 2016a; Goodman et al., 2016c)

^†^ Dissociation constants larger than 500 μM cannot be accurately determined.
