# Supplementary material for: How clustered protocadherin binding specificity is tuned for neuronal self-/nonself-recognition
Source: eLife. 2022 Mar 7;11:e72416. doi: 10.7554/eLife.72416 (PMC8901172; doi:10.7554/eLife.72416)
Supplement: Figure 3—source data 1. [file elife-72416-fig3-data1.docx]

|  | cPcdh γC4_EC1–4_  crystal form 1 | | | cPcdh γC4_EC1–4_  crystal form 2 |
| --- | --- | --- | --- | --- |
| Data collection |  | |  |  |
| Date | 02/07/2017 | | | 03/04/2017 |
| Beamline | APS 24-ID-E | | | APS 24-ID-E |
| Wavelength (Å) | 0.97918 | | | 0.97918 |
| Space group | *I*2_1_2_1_2_1_ | | | *P*2_1_2_1_2_1_ |
| *Cell dimensions* |  | |  |  |
| a, b, c (Å) | 75.220, 115.680, 194.480 | | | 51.944, 103.948, 200.592 |
| α, β, γ (°) | 90, 90, 90 | | | 90, 90, 90 |
|  | *Spherical resolution limits* | *Ellipsoidal resolution limits* | |  |
| Resolution (Å) | 40.00–3.50 (3.83–3.50) | 40–4.6/3.9/3.5 (3.84–3.51) | | 40.00–2.40 (2.49–2.40) |
| No. of reflections | 79877 (19206) | 53652 (2402) | | 240907 (16849) |
| Unique reflections | 11071 (2608) | 7545 (340) | | 42979 (4175) |
| R_merge_ | 0.381 (3.459) | 0.215 (0.830) | | 0.060 (0.438) |
| R_meas_ | 0.411 (3.722) | 0.232 (0.896) | | 0.073 (0.560) |
| R_pim_ | 0.152 (1.366) | 0.086 (0.335) | | 0.040 (0.342) |
| CC(1/2) | 0.995 (0.533) | 0.997 (0.829) | | 0.999 (0.924) |
| I/σI | 5.7 (0.8) | 8.3 (2.9) | | 13.3 (2.5) |
| Spherical completeness (%) | 99.9 (100.0) | 67.6 (12.5) | | 98.9 (92.6) |
| Ellipsoidal completeness (%) | N/A | 96.8 | | N/A |
| Redundancy | 7.2 (7.4) | 7.1 (7.1) | | 5.6 (4.0) |
|  |  |  | |  |
| Refinement |  |  | |  |
| Resolution (Å) | 40–4.6/3.9/3.5 | | | 40.00–2.40 |
| Unique reflections | 7545 | | | 42815 |
| Completeness in diffracting sphere or ellipsoid* (%) | 96.8* | | | 98.5 |
| R_work_ / R_free_ (%) | 22.3 / 27.1 | | | 19.6 / 24.1 |
| Molecules in A.S.U. | 1 | | | 2 |
| *Number of atoms* | *3242* | | | *6736* |
| Protein | 3120 | | | 6327 |
| Ligand/Ion | 122 | | | 284 |
| Water | 0 | | | 125 |
| *B-factors* | *101.24* | | | *73.39* |
| Protein | 101.03 | | | 72.99 |
| Ligand/Ion | 106.73 | | | 88.54 |
| Water | N/A | | | 59.26 |
| *R.m.s. deviations* |  | |  |  |
| Bond lengths (Å) | 0.004 | | | 0.003 |
| Bond angles (°) | 0.889 | | | 0.653 |
| *Ramachandran* |  | |  |  |
| Favored (%) | 98.80 | | | 98.92 |
| Allowed (%) | 1.20 | | | 1.08 |
| Outliers (%) | 0.00 | | | 0.00 |
| Rotamer outliers (%) | 0.29 | | | 0.57 |
| Wilson B | 77.57 | | | 51.79 |
| PDB ID | 7JGZ | | | 7RGF |

#### Figure 3­­—source data 1. X-ray crystallography data collection and refinement statistics

Values in parentheses are for the outer shell. APS, Advanced Photon Source, Argonne National Lab; A.S.U., asymmetric unit; R.m.s., Root mean square. See Figure 3­—figure supplement 1 and Methods for further details on the ellipsoidal resolution limits.
