# Supplementary material for: How clustered protocadherin binding specificity is tuned for neuronal self-/nonself-recognition
Source: eLife. 2022 Mar 7;11:e72416. doi: 10.7554/eLife.72416 (PMC8901172; doi:10.7554/eLife.72416)
Supplement: Figure 3—source data 2. [file elife-72416-fig3-data2.docx]

| **RMSDs over *trans* dimers and individual interacting domains** | γC4 crystal form 1 (intact interface) | | |
| --- | --- | --- | --- |
|  | EC1–4:EC1–4 (834 atoms) | EC1:EC4 (196 atoms) | EC2:EC3 (209 atoms) |
| γC4 crystal form 2 chain A:chain B | 2.9 Å (734 atoms) | 1.3 Å (196 atoms) | 0.9 Å (198 atoms) |
| γC4 crystal form 2 chain B:chain A | 2.9 Å (734 atoms) | 1.9 Å (194 atoms) | 1.3 Å (207 atoms) |
| *Alternate clustered Pcdhs:* |  |  |  |
| α7 dimer | 3.3 Å (801 atoms) | 2.0 Å (189 atoms) | 1.7 Å (196 atoms) |
| β6 dimer | 4.5 Å (769 atoms) | 2.5 Å (180 atoms) | 3.8 Å (200 atoms) |
| γA1 dimer | 3.5 Å (794 atoms) | 3.2 Å (191 atoms) | 2.2 Å (182 atoms) |
| γB2 dimer | 4.3 Å (802 atoms) | 1.5 Å (187 atoms) | 1.8 Å (196 atoms) |
| *Non-clustered δ1 Pcdhs:* |  |  |  |
| Human ncPcdh1 dimer | 7.0 Å (784 atoms) | 1.5 Å (139 atoms) | 2.9 Å (185 atoms) |
| *Non-clustered δ2 Pcdhs:* |  |  |  |
| Xenopus ncPcdh8.1 dimer | 3.4 Å (756 atoms) | 3.0 Å (174 atoms) | 2.4 Å (206 atoms) |
| Human ncPcdh10 dimer | 2.4 Å (785 atoms) | 2.1 Å (189 atoms) | 1.3 Å (195 atoms) |
| Human ncPcdh18 dimer | 3.1 Å (786 atoms) | 1.7 Å (157 atoms) | 2.4 Å (207 atoms) |
| Human ncPcdh19 dimer | 3.3 Å (780 atoms) | 2.0 Å (155 atoms) | 1.2 Å (187 atoms) |
| Zebrafish ncPcdh19 dimer | 2.4 Å (778 atoms) | 1.9 Å (165 atoms) | 1.2 Å (198 atoms) |

#### Figure 3—source data 2. Overall structural similarity between γC4, alternate cPcdhs, and non-clustered Pcdhs *trans* dimer structures

Table lists the pairwise root mean square deviations over aligned Cα’s (RMSDs) between the intact γC4_EC1–4_ *trans* dimer (crystal form 1) and a representative selection of available cPcdh and ncPcdh *trans* dimer structures. RMSDs between the complete EC1–4:EC1–4 *trans* dimers are given in column 2. RMSDs between individual interacting EC1:EC4 and EC2:EC3 regions of Pcdh *trans*-dimer structures are given in columns 3 and 4. The number of aligned Cα’s for each pairwise alignment is given in parentheses. The PDB codes for the aligned crystal structures are as follows: α7_EC1–5_, 5DZV; β6_EC1–4_, 5DZX; β8_EC1–4_, 5SZL; γB2_EC1–5_, 5T9T; αC2_EC1–3_, 4ZPM; γC3_EC1–3_, 4ZI8; γC5_EC1–3_, 4ZPO; ncPcdh1_EC1–4_, 6MGA; ncPcdh8.1_EC1–6_, 6VG1; ncPcdh10_EC1–4_, 6VFW; ncPcdh18_EC1–4_, 6VFR; ncPcdh19_EC1–4_, 6VFU; and zebrafish ncPcdh19_EC1–4_, 5IU9 (Goodman et al., 2016a; Goodman et al., 2016c; Rubinstein et al., 2015; Nicoludis et al., 2015; Modak and Sotomayor, 2019; Harrison et al., 2020; Cooper et al., 2016)
