# Supplementary material for: How clustered protocadherin binding specificity is tuned for neuronal self-/nonself-recognition
Source: eLife. 2022 Mar 7;11:e72416. doi: 10.7554/eLife.72416 (PMC8901172; doi:10.7554/eLife.72416)
Supplement: Figure 4—source data 1. [file elife-72416-fig4-data1.docx]

| Protein | | Oligomeric State | Dissociation Constant, K_D_ (μM) | |
| --- | --- | --- | --- | --- |
| *Cis-interacting fragments* | |  | |  |
| β1_3–6_ | | Monomer | | N/A |
| β6_1–6_ | | Tetramer | | 1.7 / 12.1^†^ |
| β9_3–6_ | | Dimer | | 35 ± 3.1 |
| γA3_3–6_ | | Dimer | | 110 ± 7.3 |
| γA4*_3–6_ | | Monomer | | N/A |
| γA9_3–6_ | | Monomer | | N/A |
| γB2*_3–6_ | | Dimer | | 80.1 ± 12.8 |
| γB5*_3–6_ | | Dimer | | 32.6 ± 4.6 |
| γB7*_3–6_ | | Dimer | | 59.0 ± 3.4 |
| αC2_3–6_-AVI | | Dimer | | 7.2 ± 1.2 |
| αC2*_2–6_ | | Dimer | | 8.92 ± 0.28 |
| α7_1–5_/γC3_6_ chimera* | | Tetramer | | 3.0 / 3.9^†^ |
| γC3*_3–6_ | | Monomer | | N/A |
| γC5_2–6_ | | Dimer | | 18.4 ± 0.24 |
|  | |  | |  |
| *Cis mutants* | |  | |  |
| γB7_3–6_ Y532G* | | Monomer | | N/A |
| γB7_3–6_ A570R | Monomer | | | N/A |
|  |  | | |  |

#### Figure 4—source data 1. Sedimentation equilibrium analytical ultracentrifugation data for *cis* SPR reagents

* Previously published data (Rubinstein et al., 2015; Goodman et al., 2016a; Goodman et al., 2017)

^†^ K_D_s of monomer-to-dimer / dimer-to-tetramer transitions from fitting the data to a tetramer model.
